# Nano-infrared imaging of metal insulator transition in few-layer 1T-TaS_2_

**DOI:** 10.1515/nanoph-2022-0750

**Published:** 2023-03-24

**Authors:** Songtian S. Zhang, Anjaly Rajendran, Sang Hoon Chae, Shuai Zhang, Tsai-Chun Pan, James C. Hone, Cory R. Dean, D. N. Basov

**Affiliations:** Department of Physics, Columbia University, New York, NY 10027, USA; Department of Electrical Engineering, Columbia University, New York, NY 10027, USA; Department of Mechanical Engineering, Columbia University, New York, NY 10027, USA; School of Electrical and Electronic Engineering, School of Materials Science and Engineering, Nanyang Technological University, Singapore 639798, Singapore

**Keywords:** phase transition, scanning near-field optical microscopy, transition metal dichalcogenides

## Abstract

Among the family of transition metal dichalcogenides, 1T-TaS_2_ stands out for several peculiar physical properties including a rich charge density wave phase diagram, quantum spin liquid candidacy and low temperature Mott insulator phase. As 1T-TaS_2_ is thinned down to the few-layer limit, interesting physics emerges in this quasi 2D material. Here, using scanning near-field optical microscopy, we perform a spatial- and temperature-dependent study on the phase transitions of a few-layer thick microcrystal of 1T-TaS_2_. We investigate encapsulated air-sensitive 1T-TaS_2_ prepared under inert conditions down to cryogenic temperatures. We find an abrupt metal-to-insulator transition in this few-layer limit. Our results provide new insight in contrast to previous transport studies on thin 1T-TaS_2_ where the resistivity jump became undetectable, and to spatially resolved studies on non-encapsulated samples which found a gradual, spatially inhomogeneous transition. A statistical analysis suggests bimodal high and low temperature phases, and that the characteristic phase transition hysteresis is preserved down to a few-layer limit.

## 1 Introduction

Transition metal dichalcogenides (TMDs) have returned to the forefront of condensed matter research in recent years, bearing the potential for novel physics and a plethora of applications [1–8]. Among these, quasi two-dimensional material 1T-TaS_2_ (T: triclinic) remains both one of the most intensely studied TMDs [9–37] and a system that continues to elude fully satisfactory answers. It has been shown to be superconducting under pressure [12, 36, 38], [39], [40] and has no long range magnetic ordering down to millikelvin temperatures, pointing to the possibility of being a quantum spin liquid [9, 17, 33], [34], [35]. Among these, one of the most noteworthy features is its rich charge density wave (CDW) phase diagram, which itself may contribute to its overall peculiar properties [10], [11], [12], [13, 26], [27], [28], [29], [30], [31], [32, 41, 42]. Of note, a sharp jump in resistivity concomitant with a low temperature CDW phase transition has been linked to a Mott insulating phase.

**Figure 1:**
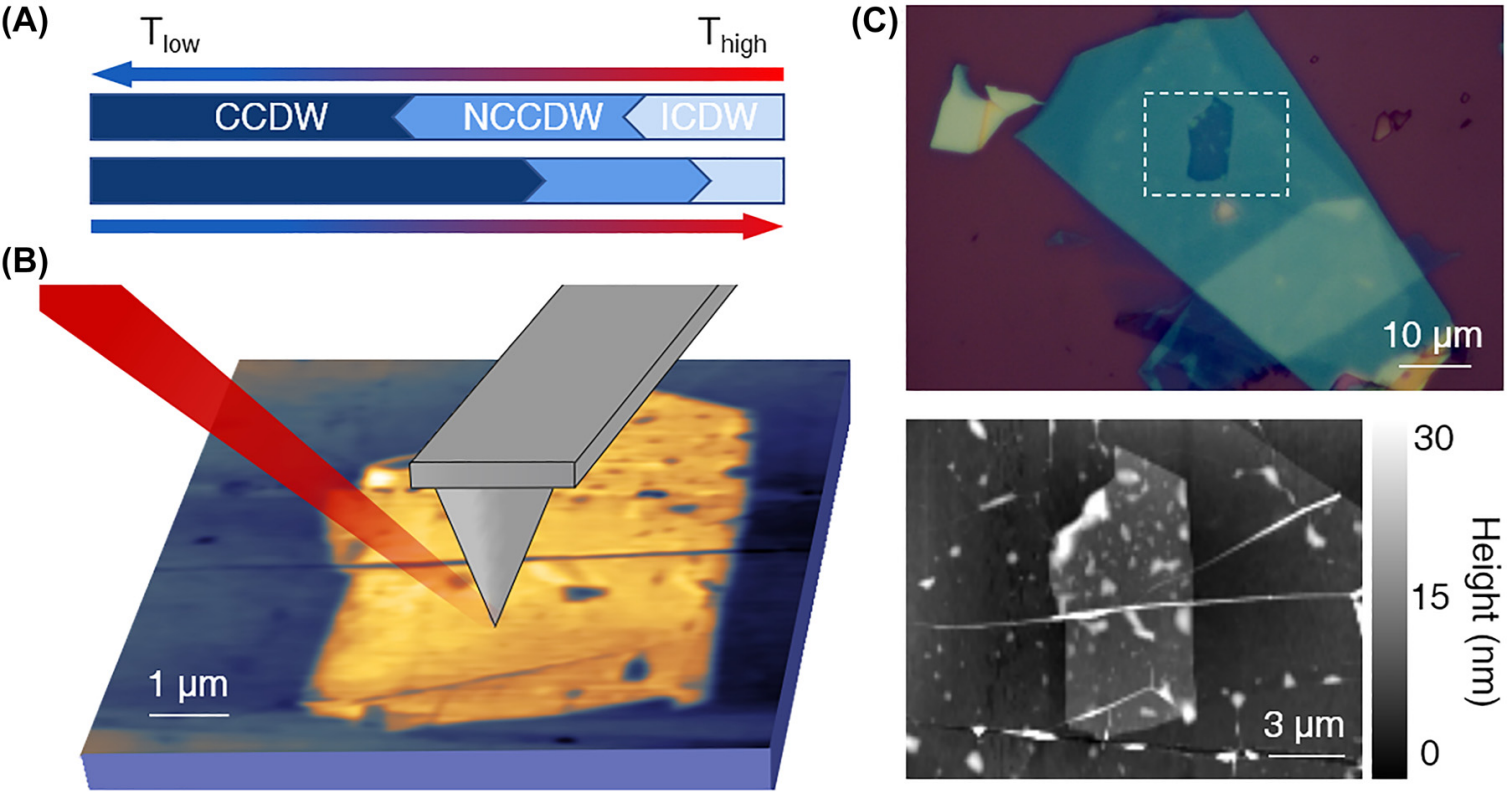
Experimental configuration of thin encapsulated 1T-TaS_2_. (A) Schematic of CDW phase transitions upon temperature variation from high (red) to low (blue) temperature and its inverse. (B) Schematic of s-SNOM experiment on 1T-TaS_2_. A laser of *λ*_0_ = 900 cm^−1^ was used. (C) Optical image of measured sample. The 1T-TaS_2_ flake is encapsulated by a layer of graphene and thin hBN, indicated by the dashed white box. (D) AFM image of the encapsulated 1T-TaS_2_ sample. The 1T-TaS_2_ flake is 5–7 nm thick.

The generally established and agreed upon charge density wave phase diagram (Figure 1A) is an initial transition from an incommensurate charge density wave to nearly commensurate charge density wave phase (ICDW and NCCDW, respectively), which occurs above room temperature near 350 K [12], [13], [14, 26, 43], [44], [45], [46], [47]. With decreasing temperature, a nearly commensurate to commensurate CDW (CCDW) phase transition occurs, with the transition temperature around 200 K in bulk samples of 1T-TaS_2_. In the bulk, each CDW phase transition has been related to a corresponding first-order transition in the resistivity, typically attributed to the formation of the hallmark of the 1T-TaS_2_ CCDW phase, where √13 × √13 Star-of-David clusters are formed by 13 Ta atoms. In each cluster, 12 Ta atoms are paired, with the spin of the remaining unpaired atom being the foundation for its quantum spin liquid candidacy [9, 17, 23, 33, 35]. In addition, the corresponding strong electron correlation is posited to drive the concomitant Mott insulator phase. Despite the many confirmations of bulk 1T-TaS_2_’s properties, questions about the electronic and structural phases in 1T-TaS_2_ with decreasing thickness remain.

## 2 Results and discussion

Here, we use scattering type scanning near-field optical microscopy [48–51] (s-SNOM) operating at cryogenic temperatures down to 50 K to study the temperature-driven phase transitions of a thin flake of 1T-TaS_2_ on a microscopic scale in real space. The spatial resolution of such measurements is largely determined by the radius of the AFM tip, on the scale of ≈20–30 nm in our measurements [49]. This allows us to probe the phase transition on the natural length scales of thermodynamic transitions, in contrast to the atomic scale of other scanning probe techniques, and with far higher resolution than far-field diffraction limited techniques. Thus, spatial variations that are otherwise inaccessible can be observed, as well as the correlation with the physical topography of the sample. A schematic of the experimental setup can be seen in Figure 1B. An incident light with wavenumber *λ*_0_ = 900 cm^−1^ is irradiated onto an atomic force microscope (AFM) tip, resulting in a strongly confined region of enhanced near-field, where the area is on the order of the tip radius, allowing for a resolution beyond the diffraction limit. The experiment is conducted in AFM tapping mode, and the AFM topography is collected simultaneously with the near-field signal. The back-scattered near field signal S is collected and demodulated at the tapping frequency to isolate it from the far field background. All data presented here have been demodulated at the third harmonic.

**Figure 2:**
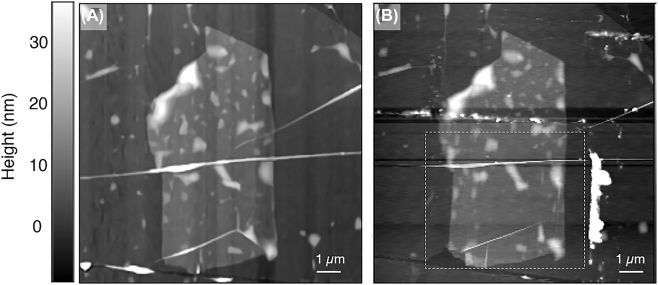
Contact cleaning of sample. (A) AFM topography of sample. (B) Same as (A), after a portion of the sample has undergone contact cleaning. The cleaned impurities are clearly seen to be pushed to the edges of the area, marked by the dotted white box.

Optical microscope and AFM topographical images of the device are shown in Figure 1C and D, respectively. The full heterostructure consists of a thin capping layer of 2 nm hexagonal boron nitride (hBN) and monolayer of natural graphene, encapsulating a thin layer of 1T-TaS_2_ of 5–7 nm in thickness. As the AFM tip is on the scale of 20–30 nm and is the primary limitation of our spatial resolution, the thin encapsulation layer is highly unlikely to impact the spatial resolution of our measurements [49]. A thick bottom layer of hBN is used to provide stability and support the overall stack. Importantly, it also isolates the device and protects it from defects on the SiO_2_/Si substrate. The 1T-TaS_2_ flake is mechanically exfoliated in a glovebox to prevent oxidation of the air unstable surface [13, 52, 53]. Due to the multi-step nature of the device fabrication, impurities are unavoidable. In order to mitigate these, contact cleaning using an AFM operating in contact mode was employed. The result of the contact cleaning can be clearly seen when comparing the topographic image of the sample before contact cleaning (Figure 2A) and after contact cleaning (Figure 2B). The lower part of the sample has a reduced number of impurities, and there is a line of ‘dirt’, which has been pushed to the edge of the cleaned area, marked in Figure 2B by a white dashed box. All measurements are focused on this area, in regions which are free of defects.

**Figure 3:**
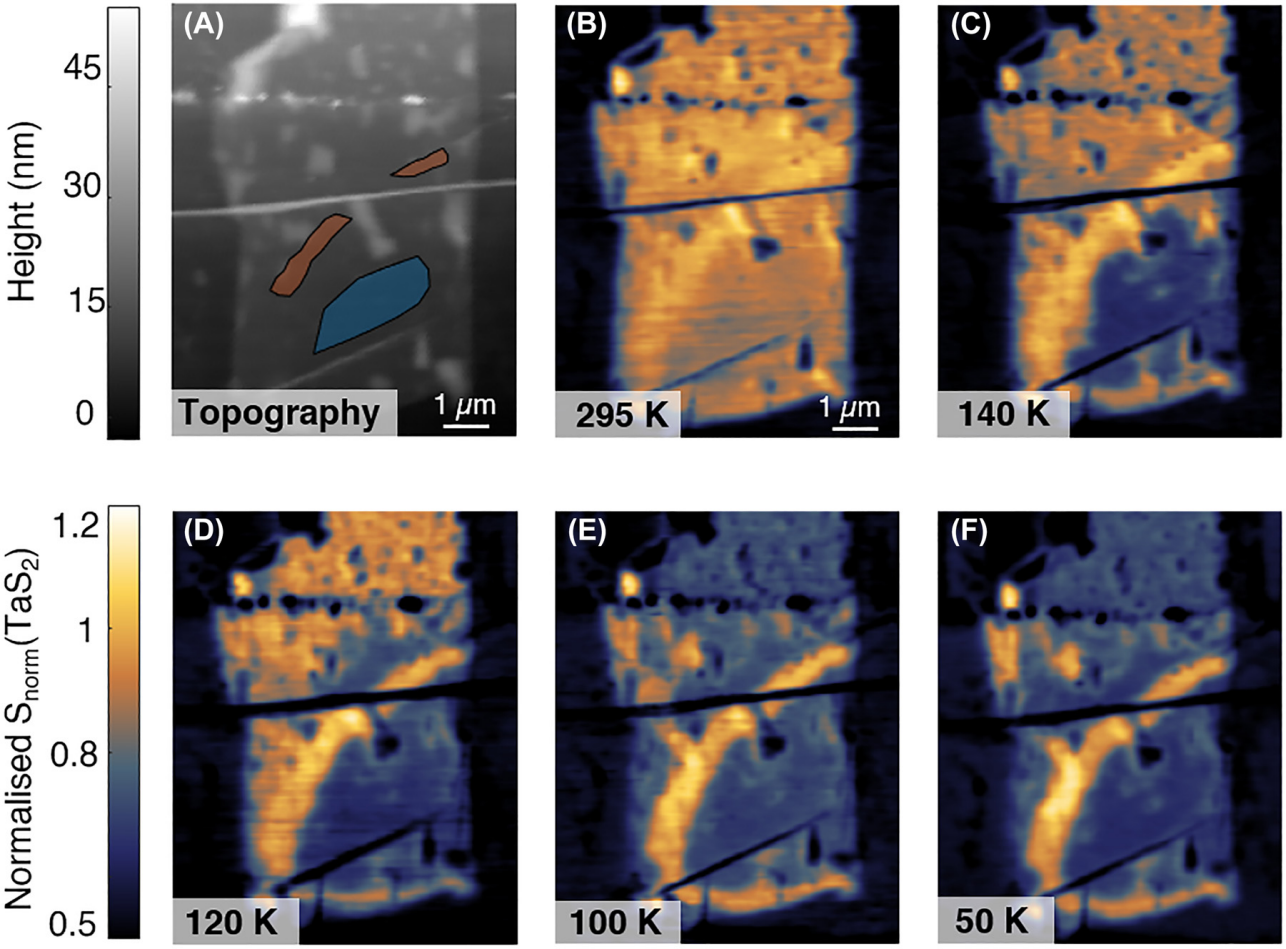
Temperature-dependent near-field images. (A) AFM topography of the sample. (B–F) Near-field amplitude images of 1T-TaS_2_ as the sample was cooled, using a laser of *λ*_0_ = 900 cm^−1^. There is a noticeable evolution in the normalised near field signal *S*_norm_(1T-TaS_2_) as 1T-TaS_2_ transitions from its metallic (bright/yellow) to insulating (dark/blue) phase near *T*_trans_ = 140 K. The normalisation is performed relative to the bright arc in the middle of the sample, as marked by the shaded orange area in (A). Statistical analysis in this work is performed on the shaded blue region in the lower right. The complete series of temperature-dependent near-field images can be found in the Supplementary.

To investigate the metal-to-insulator transition, the sample was slowly cooled from 295 K down to 50 K in our home-built cryogenic SNOM system, during which spatial near-field scans (Figure 3B–F, Figure S1) were collected along with the topography (Figure 3A) at a series of temperatures. The samples were cooled at a rate slower than 0.5 K/min and allowed to stabilise for several minutes before measurement to account for thermal drift and for the sample temperature to equilibrate. The signal was demodulated at the third harmonic S3 to separate out the near-field component. To perform temperature-dependent analysis, all near-field signals were normalised to the temperature-independent near-field signal in the region marked by orange in the topography in Figure 3A. It has been established that in SNOM measurements, metallic regions appear bright, while insulating regions appear dark (yellow and blue, respectively, in this colour scheme) [18, 54]. A visual inspection indicates that the sample is fully metallic at 295 K, indicating the expected metallicity at room temperature. An abrupt transition occurs near 140 K, where the lower (clean) region becomes insulating. The remainder of the sample undergoes a spatially gradual transition until the sample becomes fully insulating at around 50 K.

**Figure 4:**
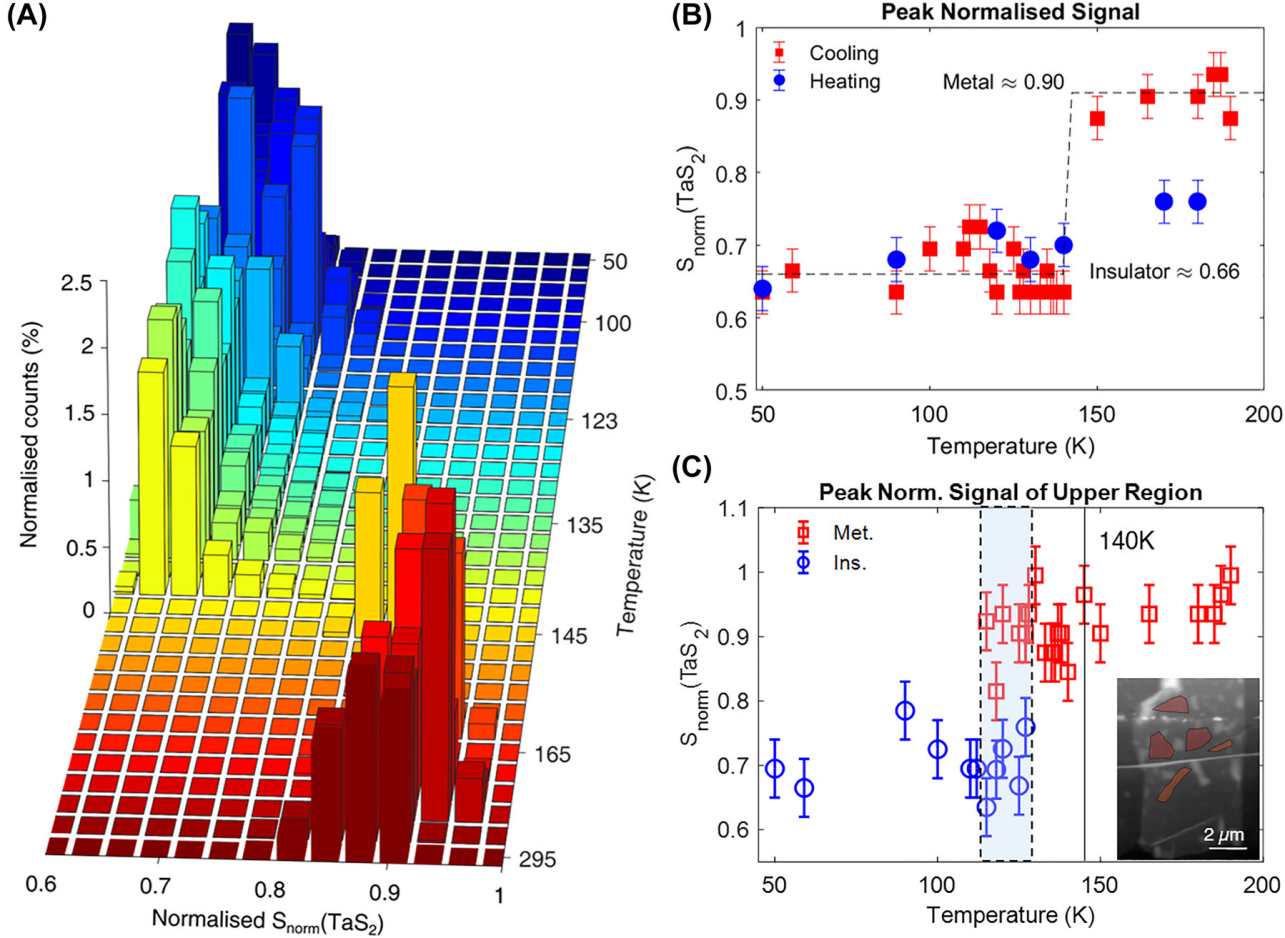
Statistical analysis of near-field response on 1T-TaS_2_. (A) Histogram of *S*_norm_(1T-TaS_2_) with decreasing temperature showing a clear bimodal distribution. A sharp transition occurs around *T*_trans_ = 140 K with no intermediate state. (B) The peak values of *S*_norm_(1T-TaS_2_) as a function of temperature, with the metallic (insulating) state marked at 90% (66%) of the normalisation value. (C) Similar to (B), but with red regions as marked in the inset, in the region of the sample with visible defects. The shaded blue dashed box indicates the temperature region where both phases coexist. The solid line marks the transition temperature in the clean lower region. Inset: AFM topography as in Figure 3A, but indicating sampled area in upper left, shaded in red.

We perform statistical analysis on the lower clean region of the sample (marked by the blue polygon in Figure 3A). This is to exclude the extrinsic effect of defects. The results are presented in Figure 4. A histogram is plotted for the normalised values of the near-field scans measured at each temperature (see Supplementary Figure S1 for near-field images at additional temperatures) and plotted as a function of temperature (Figure 4A). A clear bimodal distribution is observed, indicating a first-order transition, with one cluster centred around 0.90 of the normalisation value, and another around 0.66 of the normalisation value.

As can be visually observed in the spatial measurements (Figure 3, Figure S1), this abrupt jump in the distribution occurs at 140 K, with a change in the contrast of S3_metallic_/S3_insulating_ ≈ 1.36 over a 5 K change in temperature (Figure S1). Although there is some variation in peak location, no clear temperature dependence is observed. The peak locations of the normalised values for each temperature are plotted in Figure 4B. The cooling curve, for which the histogram is plotted in Figure 4A, is marked in red. Measurements taken upon heating of the sample are marked by the blue squares (the near-field images can be found in Supplementary Materials, Figure S2). Dashed lines are provided as a guide to the eye. The metallic and insulating values of 0.90 and 0.66, respectively, are obtained by a linear fit to the peak values above and below *T* = 140 K, respectively. While experimental constraints prevented a complete measurement of the thermal cycle, enough heating data were obtained to show that the sample remains insulating to at least 170 K, indicating temperature-dependent hysteresis in this thin flake of 1T-TaS_2_.

The clear separation of the peaks in the bimodal distribution and hysteresis of the near-field signal in the cleaned, isolated region of our microcrystal contrasts with some earlier works on thin samples of 1T-TaS_2_ [14, 26, 55]. However, transport measurements capture a global resistivity of the entire sample and cannot account for spatial variations. Furthermore, some of these earlier works of thin 1T-TaS_2_ were done on samples exposed to air, while measurements done on samples protected from ambient conditions have shown that this transition can persist in the few layer limit [13, 24]. We attribute the difference in our results to the sample encapsulation and the local measurement being performed only on the clean region, minimising the effect from defects. The protection of the sample from substrate defects may also be important, as the phase transition in 1T-TaS_2_ can be affected by substrate properties [56, 57]. We would like to highlight that in contrast to Ref. [18], the sample studied here is fully isolated from the substrate, in addition to being thinner. Notably, Ref. [18] found a temperature dependent shift in the peak centre of *S*_norm_(1T-TaS_2_), particularly in the metallic state, as well as temperature regimes for which both the metallic and insulating state co-exist with a smooth transition boundary between the regions. From the spatial near-field images, (Figure 3A–E, Figures S1 and S2), a significant spatial variation of the transition exists in regions with more defects. To contrast with the results in the cleaned, isolated region where there was no spatial inhomogeneity, in Figure 4C, we performed a similar statistical analysis as in 4B, albeit in this defect-rich region, and excluding the defects themselves (red regions in inset). In the temperature region between ≈110 K and 130 K, we can observe the existence of both the metallic and insulating mode (dashed box shaded in blue). Furthermore, there is a temperature-dependent shift in the spectral weight between these two modes (see Figure S7), reminiscent of what was observed in Ref. [18]. It is less clear if there is any temperature dependence of the metallic and insulating peaks in *S*_norm_. However, due to uncertain nature of the defects, we can only hypothesise that external effects, potentially strain, can lead to inhomogeneous mixed phases.

Recent out-of-plane measurements of electrical properties also suggest significant in and out of plane anisotropy. Such effects may be negligible in bulk and bulk-like samples but cannot be ignored in thin samples such as the one studied here. Indeed, a recent transport and DFT + U study lends support to the importance of dimensionality [24]. The lower transition temperature is in line with thickness dependence studies, which found that decreasing the layer number decreases the transition temperature of the NCCDW-CCDW transition [24, 26]. Simulations of the expected near-field signal contrast between the metallic and insulating states were performed following Ref. [18, 50]. While a contrast ratio of S3_metallic_/S3_insulating_ ≈ 1.36 was observed in measurement, the simulation reflected a lower contrast with a calculated ratio ≈1.1 (Figure S4 and inset). A previous study using a similar theoretical simulation on a moderately thicker sample also finds an underestimation of the near-field contrast at a different measurement frequency [18]. This discrepancy may be a further reflection of physical parameters the calculation is unable to capture, such as a distortion of the lattice structure, or modification of the band structure [24, 27].

To summarise, we clearly observe the metal-to-insulator transition corresponding to the NCCDW-CCDW transition and the associated hysteresis. By using high resolution near-field optical measurements, we can minimise the extrinsic effects of defects by focusing on a local, clean area. Encapsulation of the sample from both ambient and substrate conditions further reduced defects. The observed sharp transition is in contrast to previous works where it was either not observed or a more gradual increase in the resistivity was measured [13, 14, 26, 55]. Furthermore, the study presented here provides an accurate spatial mapping of the metal-to-insulator transition of thin-flake 1T-TaS_2_. The difference in spatial variation with temperature evolution and the bimodality of the insulating and metallic states in this work further highlight the need to consider the role of sample encapsulation and sample thickness [18]. Such spatial variation can account for the broader and less pronounced transition of the resistivity difference in transport measurements between the NCCDW and CCDW state in thin samples [13, 14, 26]. Future work studying thickness dependence of locally clean samples with nanoscale resolution can help clarify the role of dimensionality and interlayer interactions.

## 3 Methods

### 3.1 Device preparation

The device is an all van der Waals stack consisting of a few-layer 1T-TaS_2_-graphene heterostructure encapsulated by hexagonal boron nitride (hBN) on the top and bottom. Thin TaS_2_ is air-sensitive and is, therefore, exfoliated inside a nitrogen-filled glovebox to prevent any degradation and oxidation. A thin flake is mechanically exfoliated using Nitto tape onto a 285 nm SiO_2_/Si substrate. The hBN and graphene is mechanically exfoliated onto the 285 nm SiO_2_/Si substrate using scotch tape under ambient conditions and loaded into the glovebox for device assembly. The device is stacked using the dry-pick up technique. The thin top hBN is picked up using a polydimethylsiloxane (PDMS) stamp coated with a polymer polycaprolactone (PCL). Next, this top hBN is used to pick up the graphene, 1T-TaS_2_ and bottom hBN stack. The top and bottom layers of hBN protect the 1T-TaS_2_ from disorders, defects from both the substrate and environment and also prevent oxidation and degradation. After picking up the entire stack, the device is transferred onto a 285 nm SiO_2_/silicon chip by melting the PCL polymer at 75 °C. The stack is then cleaned by immersing the stack in hot acetone at 75 °C for 30 min with a subsequent acetone and IPA rinse.

### 3.2 Near-field measurements

All measurements were performed using a home-built cryogenic scattering type scanning near-field optical microscope based on a tapping-mode atomic force microscope in an ultra-high vacuum environment on the order of 10^−10^ Torr. A commercial Arrow™ tip was used, with tapping frequency near 75 kHz. A CO_2_ laser was used with incident light frequency of 900 cm^−1^, focused onto the AFM tip. The back-scattered light was collected with pseudo-heterodyne interferometric detection. The detected signal was demodulated at the third harmonic to obtain the near-field signal by minimising the background contributions of the scattered light.

## Supplementary Material

Supplementary Material Details
