# Supplementary material for: Nano-infrared imaging of metal insulator transition in few-layer 1T-TaS2
Source: Nanophotonics. 2023 Mar 24;12(14):2841–7. doi: 10.1515/nanoph-2022-0750 (PMC11501826; doi:10.1515/nanoph-2022-0750)
Supplement: Supplementary file 1 — Supplementary Material Details [file j_nanoph-2022-0750_suppl_001.pdf]

Nano-infrared imaging of metal insulator transition in few-layer 1T-TaS_2

Supplementary Information

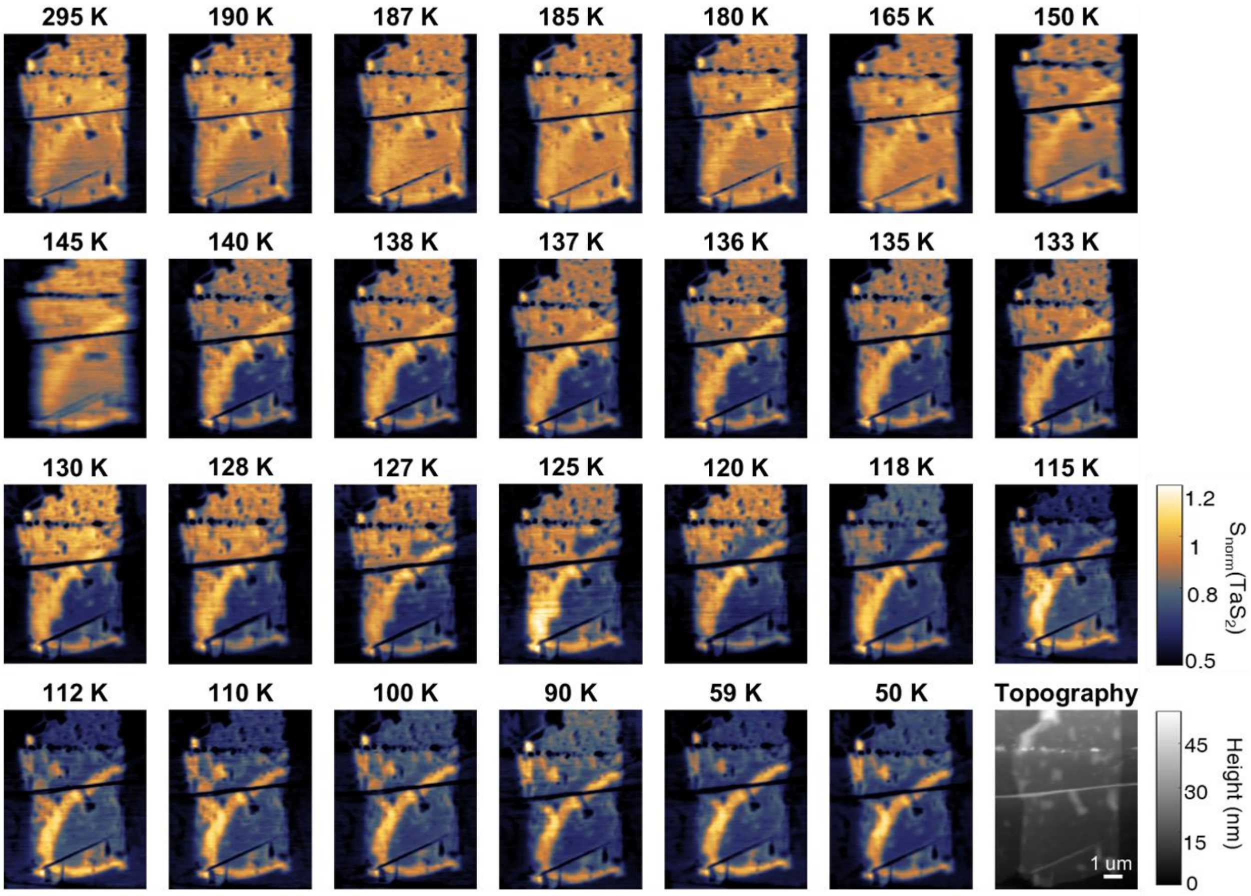

Supplementary Figure 1. Near field amplitude images of 1T-TaS_2 taken upon cooling. The scale bar is the same for all images.

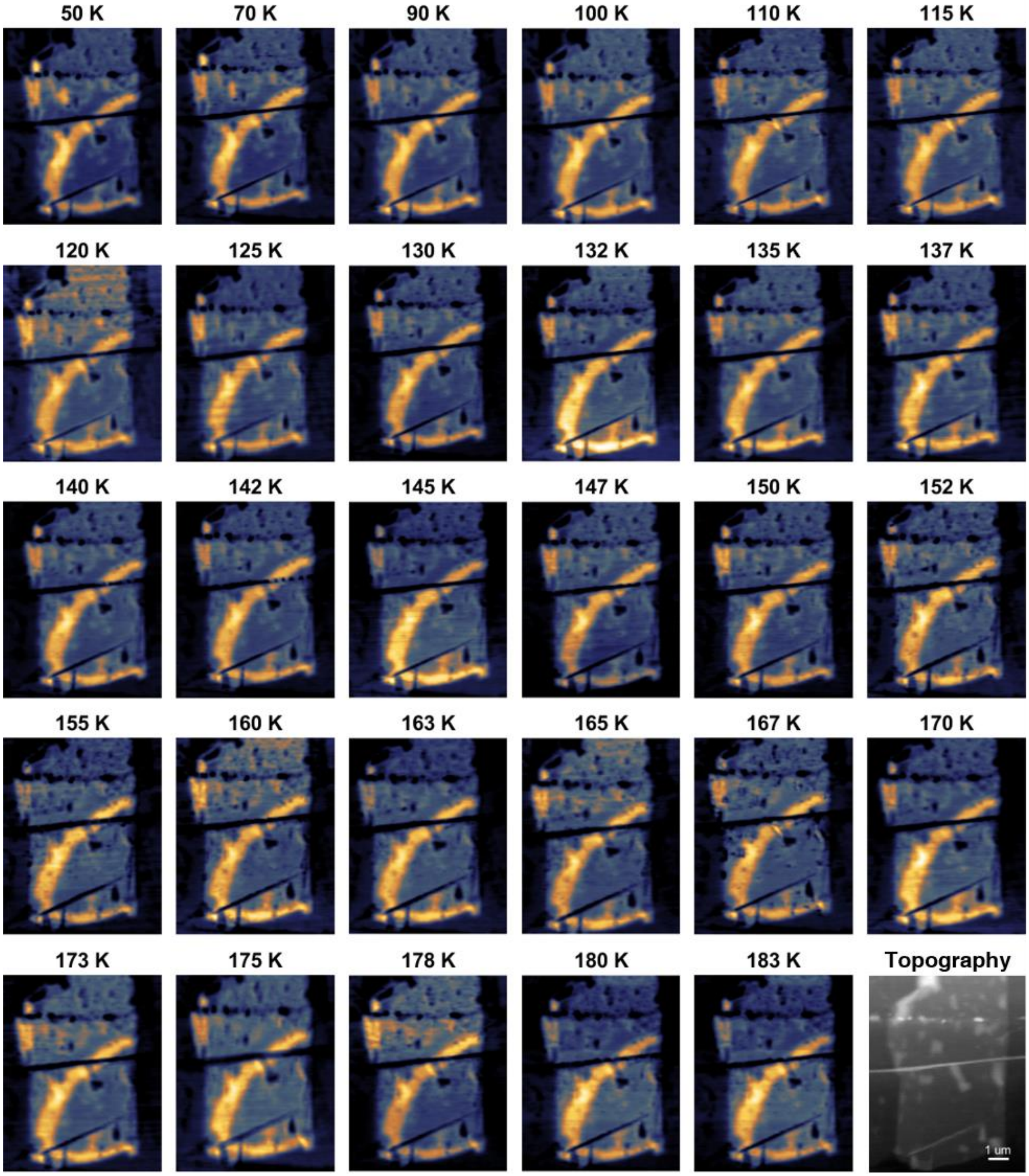

Supplementary Figure 2. Near field amplitude images of 1T-TaS_2 taken upon heating. The scale bar is the same for all images as in Fig. S1. No clear transition is seen up to 183K.

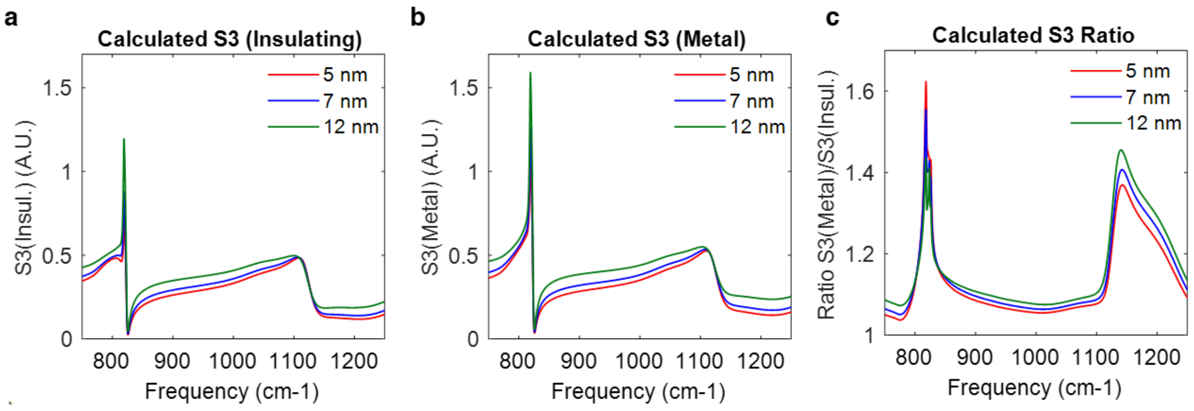

Supplementary Figure 3. Calculated near-field signals at **a)** room temperature in the metallic state, and at **b)** low temperature in the insulating CCDW state, and **c)** the ratio for samples with 1T-TaS_2 flakes of thicknesses of 5nm, 7nm, and 12nm. Arbitrary units are used in a and b. All calculations in main text assume a thickness of 7nm.

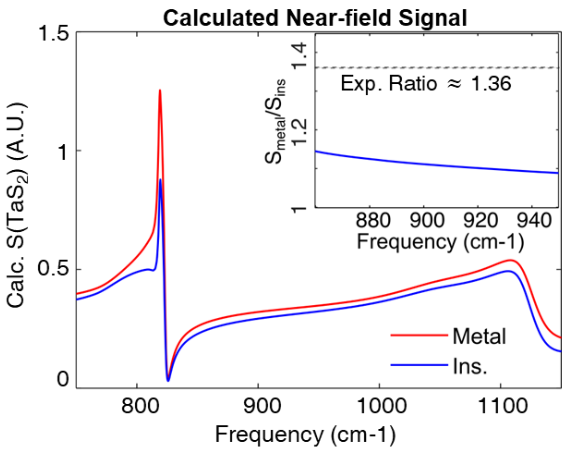

Supplementary Figure 4. Calculated near-field signal normalised to gold S(1T-TaS_2)/S(Au) of the metallic and insulating state over an extended frequency range. Inset: contrast of the near-field signal between the metallic and insulating state. The experimentally measured ratio $S_{metal}/S_{insulator} \approx 1.36$ is marked with a dashed line.

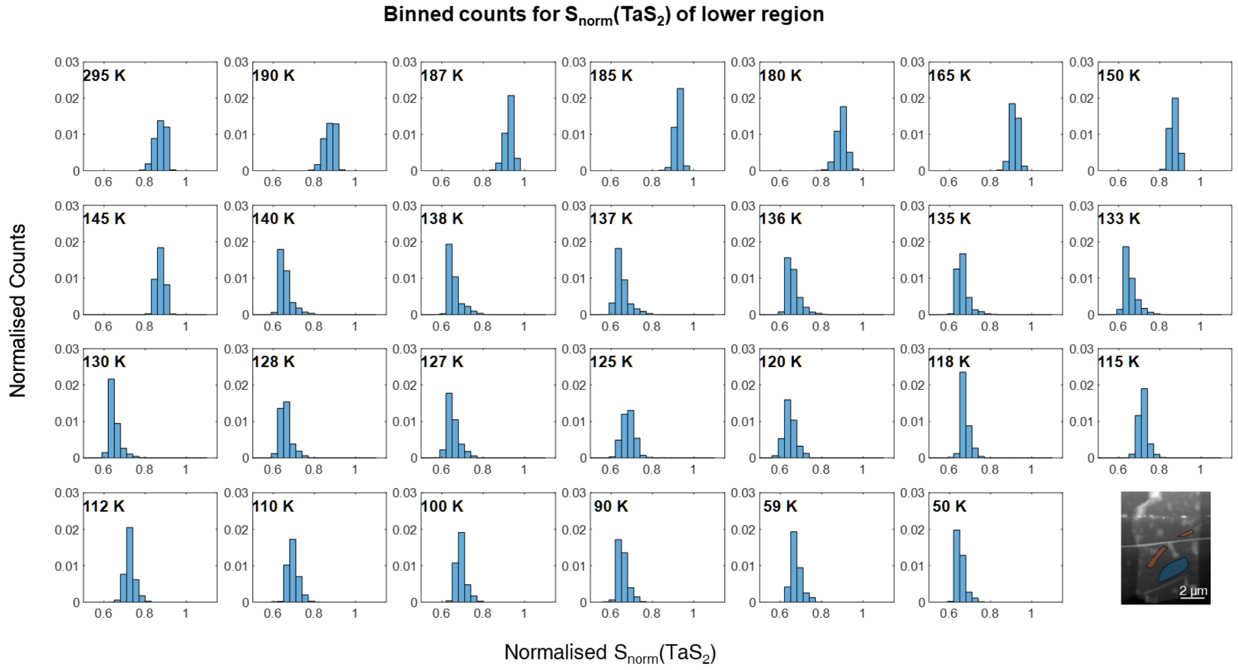

Supplementary Figure 5. Individual histogram plots for the lower region marked in blue in the lower right panel, as also presented in Fig. 4A.

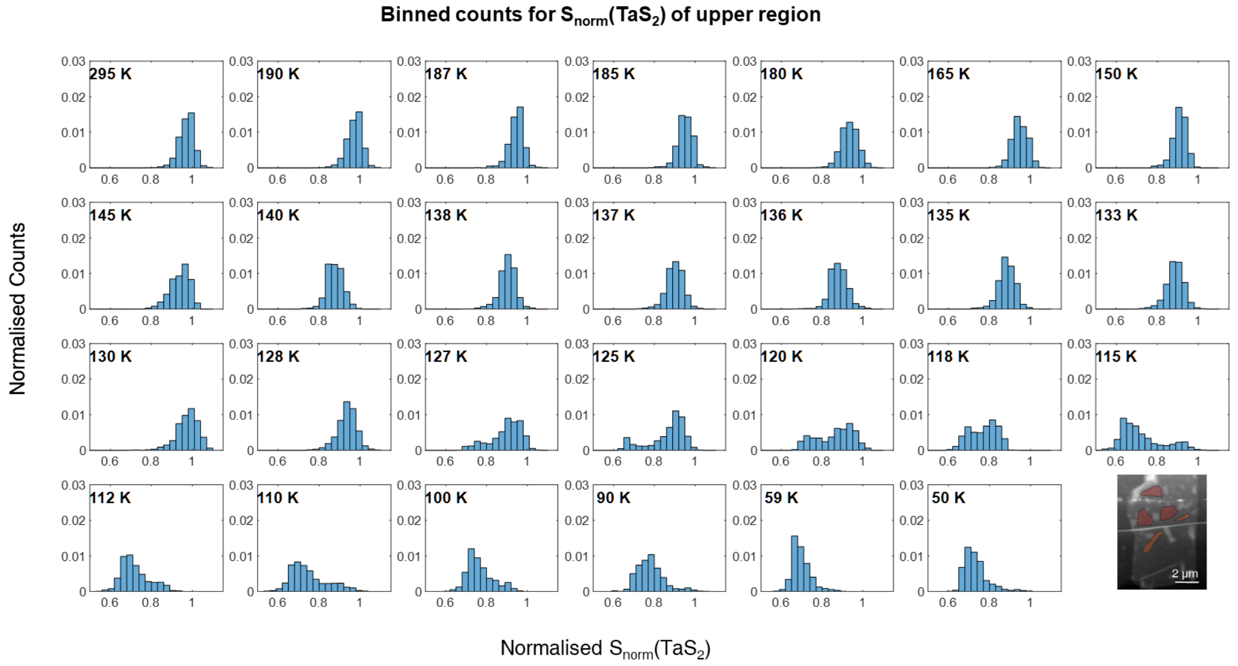

Supplementary Figure 6. Individual histogram plots for the upper region marked in red in the lower right panel.

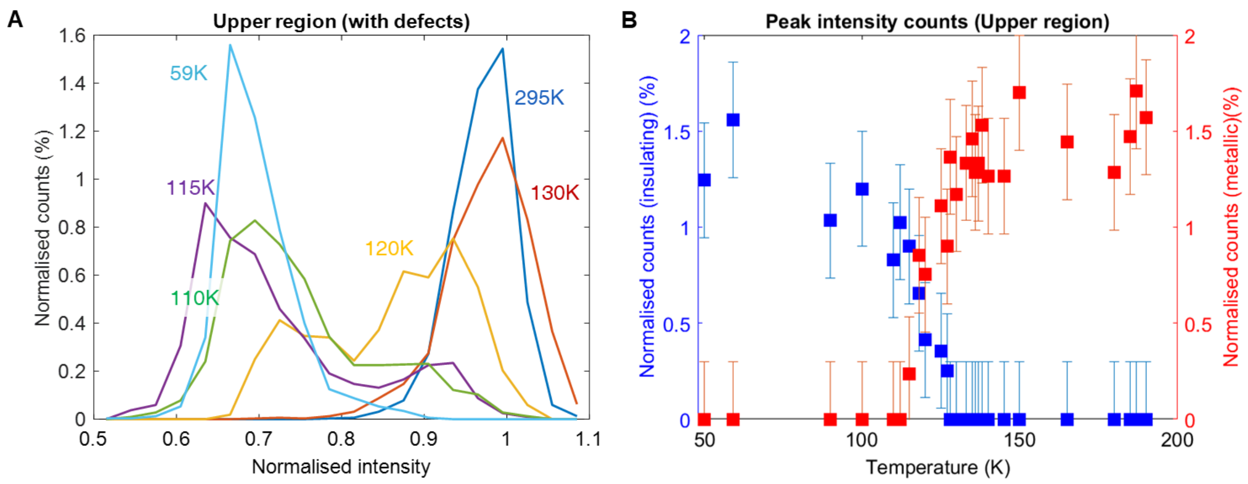

Supplementary Figure 7. **A**, Normalised counts of S_{norm} for the upper region as marked in red in Figure S6. With decreasing temperature, there is a shift in the fraction of metallic to insulating regions, but it is unclear if there is a temperature dependence of the peak center. **B,** The normalised counts of the metallic (red) and insulating (blue) peaks for the defect rich upper region.
